# Reassessing the role of urban green space in air pollution control

**DOI:** 10.1073/pnas.2306200121

**Published:** 2024-01-29

**Authors:** Zander S. Venter, Amirhossein Hassani, Erik Stange, Philipp Schneider, Núria Castell

**Affiliations:** ^a^Norwegian Institute for Nature Research, Oslo 0855, Norway; ^b^The Climate and Environmental Research Institute NILU, Kjeller 2027, Norway

**Keywords:** vegetation, urban planning, green infrastructure, ecosystem service, public health

## Abstract

The assumption that vegetation improves air quality is prevalent in scientific, popular, and political discourse. However, experimental and modeling studies show the effect of green space on air pollutant concentrations in urban settings is highly variable and context specific. We revisited the link between vegetation and air quality using satellite-derived changes of urban green space and air pollutant concentrations from 2,615 established monitoring stations over Europe and the United States. Between 2010 and 2019, stations recorded declines in ambient NO_2_, (particulate matter) PM_10_, and PM_2.5_ (average of −3.14% y^−1^), but not O_3_ (+0.5% y^−1^), pointing to the general success of recent policy interventions to restrict anthropogenic emissions. The effect size of total green space on air pollution was weak and highly variable, particularly at the street scale (15 to 60 m radius) where vegetation can restrict ventilation. However, when isolating changes in tree cover, we found a negative association with air pollution at borough to city scales (120 to 16,000 m) particularly for O_3_ and PM. The effect of green space was smaller than the pollutant deposition and dispersion effects of meteorological drivers including precipitation, humidity, and wind speed. When averaged across spatial scales, a one SD increase in green space resulted in a 0.8% (95% CI: −3.5 to 2%) decline in air pollution. Our findings suggest that while urban greening may improve air quality at the borough-to-city scale, the impact is moderate and may have detrimental street-level effects depending on aerodynamic factors like vegetation type and urban form.

## Significance

Our findings indicate that the relationship between urban vegetation and air quality is more complex than previously thought. While urban greening has other positive health outcomes for residents, our study suggests that it may not be an efficient abatement measure for air pollution. Although we found minor amelioration effects of vegetation at the borough to city scale, street-level vegetation can act to exacerbate air pollution. Reducing anthropogenic emissions instead of urban greening should be the primary focus for improving air quality.

Exposure to air pollution currently results in more deaths than malaria, tuberculosis, and HIV/AIDS combined (1). Particulate matter with a diameter of less than 2.5 µm (PM_2.5_) is estimated to cause up to 10 million excess deaths globally (2, 3). Consequently, the World Health Organization has identified air pollution as the single biggest environmental threat to human health (4). Given that over 70% of the global health burden from air pollution is attributable to anthropogenic emissions (3, 5), the majority of policies to improve air quality are focused on cutting emissions (6). Examples of emission abatement actions include cleaner energy production, efficient industrial smokestacks, reduced reliance on diesel vehicles, and sustainable agriculture practices. An alternative set of strategies involve removing or remediating air pollution after it has been emitted, through both active and passive abatement technologies (7). While active abatement technologies such as physical-chemical filters dominate the literature on indoor air pollution (8), passive abatement methods—such as utilizing vegetation—are most prevalent in the literature on outdoor or ambient air pollution (9).

The assumption that urban vegetation, also referred to as green space or green infrastructure, can improve air quality is widely held in the public health (10), urban planning (11), and ecosystem service (12) literature. Popular media (e.g., ref. 13) and even international standards and policy frameworks such as the UN System of Environmental-Economic Accounting propose vegetation as a nature-based solution to decrease air pollution (14). The primary mechanisms through which air pollution may be reduced by vegetation include deposition and dispersion (15). Deposition occurs when air pollutants adsorb to vegetative surfaces, whereas dispersion involves the dilution of air pollutant concentrations due to aerodynamic effects induced by vegetation structures. Dispersion effects outweigh deposition by an order of magnitude (16). However, the mechanisms of dispersion effects can also increase local air pollution concentrations—depending on the structure of the vegetation (e.g., height, leaf density), site context (e.g., street canyon geometry, distance to emission source), and prevailing weather (e.g., wind speed and direction) (11, 16). For instance, dense tree canopies can reduce ventilation in street canyons, and porous vegetation barriers in open-road settings can exacerbate roadside air pollution concentrations (9, 17). Vegetation can also produce volatile organic compounds (VOC) that, in cases like Los Angeles, contribute to a quarter of the secondary organic aerosol on hot days (18). Although there is evidence that vegetation can ameliorate air pollution under the right circumstances, there is ample experimental and modeling evidence to the contrary (9, 10, 17). The conflict in the scientific literature is perhaps why the blanket assumption that urban green space reduces air pollution remains widespread in popular discourse. One possible explanation for the knowledge gap is the local nature of experimental and modeling studies that are not necessarily generalizable across broader spatial scales.

Here, we use regional observational data to test the hypothesis that changes in ambient air pollution are associated with changes in surrounding urban green space. We use an established network of air quality stations over Europe (from the European Environmental Agency; EEA) and the United States (from the Environmental Protection Agency; EPA) to derive annual time series of NO_2_, PM_10_, PM_2.5_, and O_3_ concentrations between 2010 and 2019. Green space changes around each air quality station are measured using (1) the normalized difference vegetation index (NDVI) and fractional tree cover from moderate resolution satellites and (2) visual interpretation of very high-resolution aerial imagery at a sub-set of air quality stations. Using linear mixed-effects models, we estimate the association between green space and air quality after controlling for changes in anthropogenic emissions and climate.

## Results and Discussion

### Air Pollution Changes.

**Fig. 1.**
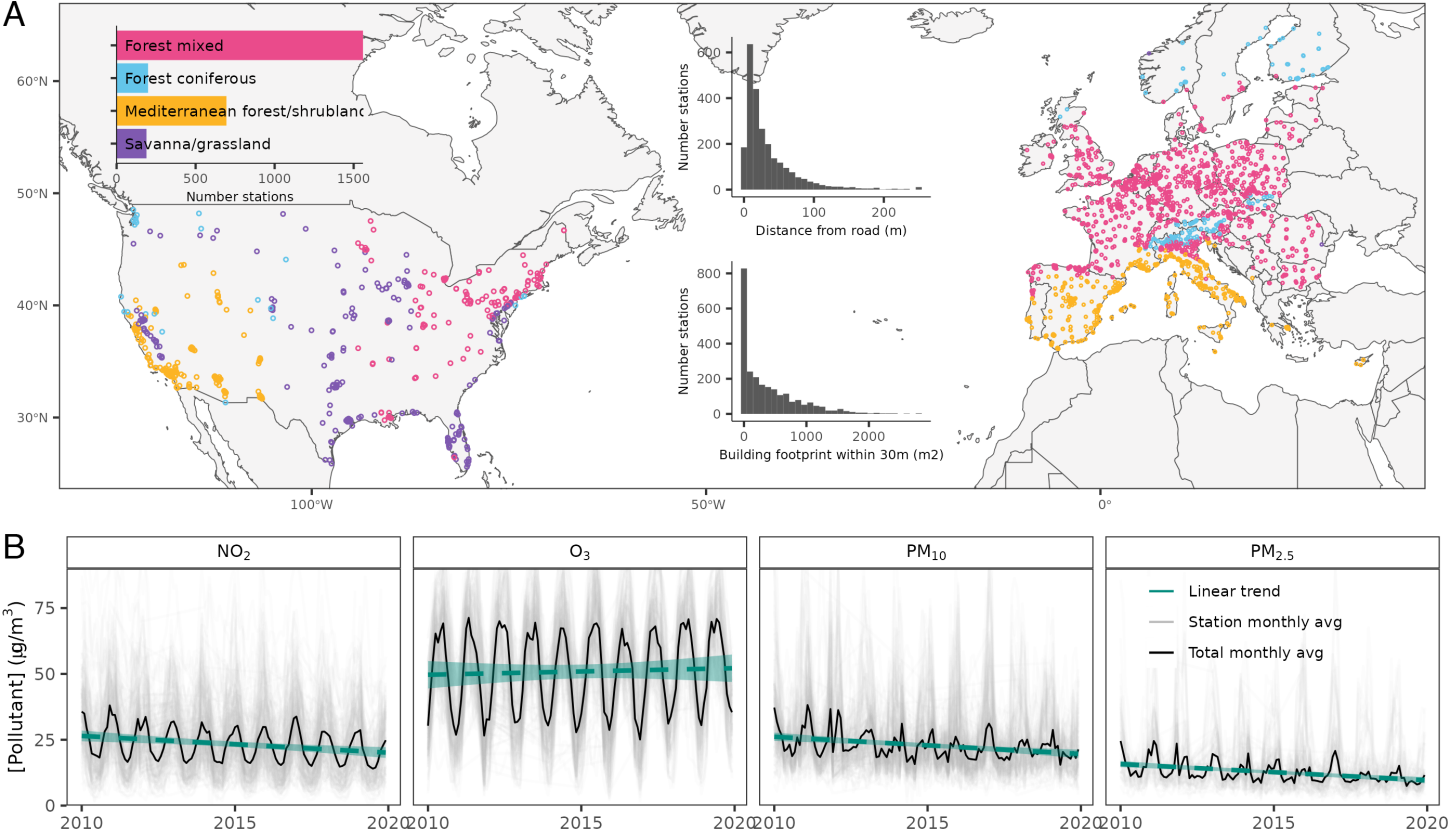
Distribution of the air quality–monitoring stations across biomes in Europe (n = 2,127) and the United States (n = 488) (*A*). *Inset* histograms show the proximity of stations to roads and the building footprint within 30 m. Air pollutant time series along with linear trends are shown in (*B*).

Data from 2,615 air quality stations revealed declines in ambient NO_2_ (−2.9 ± 0.06% y^−1^ 95% CI), PM_10_ (−2.93 ± 0.06% y^−1^), and PM_2.5_ (−3.6 ± 0.1% y^−1^) between 2010 and 2019 (Fig. 1). In absolute terms, this equates to an average decline in concentration of 7.95 (NO_2_), 4.6 (PM_10_), and 3.83 µg m^−3^ (PM_2.5_). In contrast, O_3_ increased in concentration by 0.5 ± 0.08% y^−1^. Declines were relatively consistent across the United States and Europe, although increases in pollutant concentrations were evident in Southern Europe and Western United States, especially for PM (*SI Appendix*, Fig. S1). The changes in air quality found here are broadly consistent with the trends reported in earlier studies using both the regulatory monitoring station networks as well as satellite instruments (e.g., refs. 19–22). The trend values found here are slightly higher in magnitude than a very recent paper studying European air quality trends for the 2005 to 2019 period after correcting for the impact of meteorology (23). The latter found median trends of −2.1% y^−1^ for NO_2_, −2.2% y^−1^ for PM_10_, and −2.2% y^−1^ for PM_2.5_ for urban/suburban stations. It should be noted that in absolute terms the reductions found in this study as well as in Walker et al. (23) (ca. −4 to −13 µg m^−3^ for NO_2_, −7 to −13 µg m^−3^ for PM_10_, and −4 to −7 µg m^−3^ for PM_2.5_, all for the 14-y period) are substantially smaller in magnitude compared to reductions found for example in China [ca. −8.2% y^−1^ and ca. −20 µg m^−3^ overall for PM_2.5_ for 2013 to 2017, (24)]. However, trends can vary significantly with the selected study period as well as the applied methodology and are thus challenging to directly compare between different studies.

### Drivers of Air Pollution Change.

To a large extent, the broad-scale improvements in air quality found here are a direct result of successful policy-driven emissions reductions that have been carried out in both Europe and the United States over the study period (25). The same responses to emission regulations have recently been observed in China (24). However, our approach explored whether local-scale processes such as vegetation change and climate dynamics might explain some of the spatial and temporal variation in air quality changes. We first stratified our change analysis by biome, given that biome-specific vegetation types and dominant climate are important mediators of vegetation’s effects on air quality. Stations falling within the forest biomes exhibited greater declines compared to those in the Mediterranean shrubland and savanna/grassland biomes, particularly for PM (*SI Appendix*, Fig. S1). This is possibly due to the long-range aerosol transport of dust and smoke, which is more prevalent in arid environments such as the Mediterranean. It is also possibly due to the greater deposition and dispersion capacity of forest vegetation compared to Mediterranean shrubland. We found that, while PM_10_, PM_2.5_, and NO_2_ were positively correlated with each other at the station level, O_3_ showed greater variability and was most often negatively correlated with the other air pollutants (*SI Appendix*, Fig. S2). This is due to nonlinear chemical interactions between NO_x_ and VOCs (26). For instance, the emission decline of NO_x_ (=NO + NO_2_) can lead to reduced local titration of O_3_ (reaction of NO with O_3_).

We explored the association between changes in green space and air pollution using linear mixed models that controlled for the effects of local emissions and climate changes. Two characterizations of vegetation surrounding air pollution stations were derived from satellites including green space in general and tree cover in particular. NDVI from the Landsat satellites at 30 m resolution was used to quantify total green space because it is a widely used proxy for capturing dynamics in vegetation cover and productivity (27). We used a product developed by the United States Geological Survey from the Moderate Resolution Imaging Spectroradiometer (MODIS) at 250 m resolution to quantify changes in tree cover. Changes in total green space and tree cover were aggregated within circular buffer zones surrounding air pollution stations with radii ranging from 15 to 16,000 m. To aid interpretation, we categorized spatial scales of street-level (15 to 60 m), borough-level (120 to 1,000 m), and city-level (2,000 to 16,000 m). Due to the limited spatial resolution in MODIS data, we were not able to characterize changes in tree cover at street-level, yet we were able to using Landsat NDVI.

**Fig. 2.**
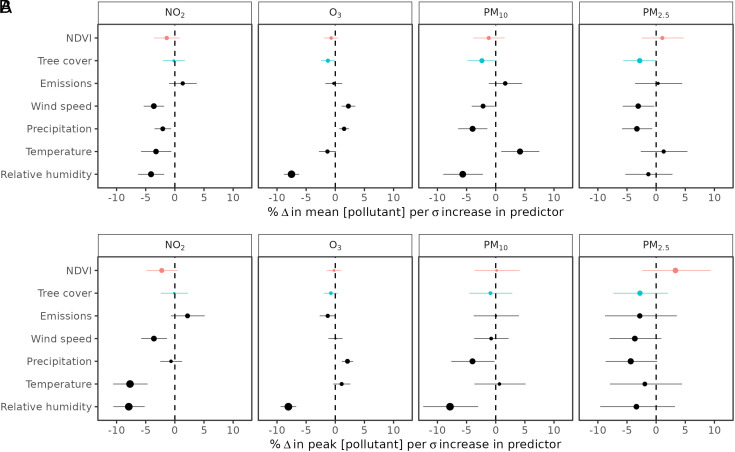
Empirical estimates of the association between annual mean (*A*) and maximum (*B*) air pollutant changes, and changes in green space, emissions, and climate predictor variables. Estimates and 95% CI are indicated with points and error bars and are expressed as percentage changes in air pollutant concentrations per SD (δ) increase in the predictor variable. The size of the point indicates the magnitude of the effect.

The effect of changes in total green space on mean annual and peak air pollution changes was negligible when aggregated across spatial scales (Fig. 2). Please note that “effect” here refers to the statistical direction and magnitude of association between explanatory and response variables and should not be interpreted as indicating causality. When averaged across pollutants, a one SD increase in total green space was associated with a 0.8 ± 2.7% decrease in pollutant concentrations. Despite the lack of an overall green space effect, increases in tree cover in particular were associated with declines in O_3_, PM_10_, and PM_2.5_. When averaged across pollutants, a one SD increase in tree cover was associated with a 1.4 ± 2.5% decrease in pollutant concentrations. The largest tree cover effect size was for PM where, on average, one SD increase in percentage tree cover resulted in a 2.7% decline in pollutant concentrations, equating to 0.27% y^−1^ (Fig. 2*A*). The effect sizes of green space were smaller in magnitude than climatic drivers. For example, one SD increase in relative humidity was associated with an 8% decrease in peak annual NO_2_, O_3_, and PM_2.5_ (Fig. 2*B*). Humidity, wind speed, and precipitation also had negative associations with all pollutants except for O_3_.

Given the larger effect sizes from climatic drivers, meteorological dynamics may dominate the marginal effects that green space may have when aggregating across spatial scales. Our finding aligns with the balance of the evidence from observational studies. For instance, in a similar analysis across 31 provincial capital cities in China, He et al. (28) found that meteorology explains 70% of the variance in urban pollutant concentration reduction. In another study in China between 2014 and 2019, wind speed and precipitation were significantly negatively correlated with urban air pollution concentrations at most of the 896 stations (29). The general consensus is that traffic-induced pollution is more prevalent in stagnant, cold weather conditions in urban areas. However, the response of PM to wind can vary depending on wind speed and street canyon orientation and form–as well as particle size. For example, coarse particle deposition on vegetation occurs more efficiently at high wind speeds, while ultrafine particles accumulate more slowly (15). Apart from one study in India finding negative correlations between PM and humidity (30), reviews of the literature show that the effect of humidity on air quality is understudied (11). Our findings suggest a negative relationship between humidity and air pollution, which might be due to an increased rate of absorption of particulates in the atmosphere at elevated humidity. Humidity is also highly correlated to precipitation and this combination may act as a natural scrubber through increased particle deposition on all surfaces.

In our spatially aggregated models, we explored both annual mean (Fig. 2*A*) and annual peak (Fig. 2*B*) pollution concentrations. The effect sizes of green space, emissions, and climate variables across pollutants were similar when considering changes in annual mean versus peak concentrations (Fig. 2*B*). This suggests that the association between vegetation and air pollution is unaffected by the severity of pollutant concentrations within the years considered. Hereafter, we focus on associations with mean annual pollutant concentrations which is often used a proxy for cumulative exposure in epidemiology studies (2, 31).

**Fig. 3.**
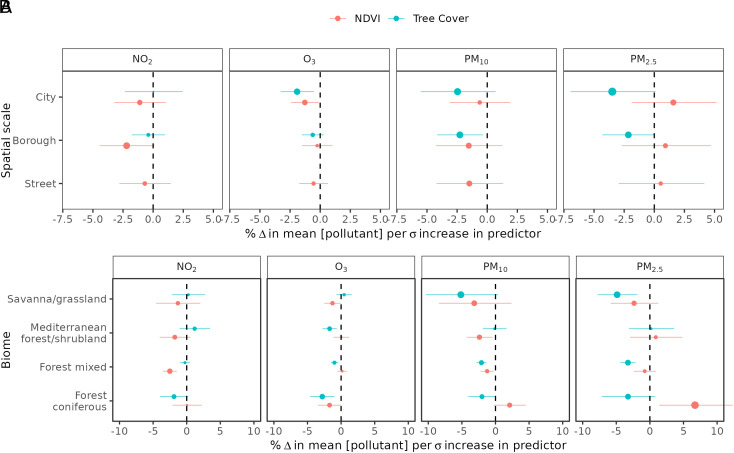
Empirical estimates of the association between annual mean air pollutant changes, and changes in green space predictor variables including NDVI (total green space) and fractional tree cover. Separate models and effect sizes were estimated for each spatial scale over which green space was aggregated (*A*) and each biome within which stations were located (*B*). Estimates and 95% CI are indicated with points and error bars and are expressed as percentage changes in air pollutant concentrations per SD (δ) increase in the predictor variable. The size of the point indicates the magnitude of the effect.

The effect of green space varied over spatial scales (Fig. 3*A*). Total green space exhibited a negative association with NO_2_ at borough scales, but the effect was minimal at street and city scales. The negative effect of tree cover on O_3_, PM_10_, and PM_2.5_ was enhanced with increasing spatial extent from borough to city scales. Fine PM_2.5_ exhibited divergent responses to green space at the city scale (Fig. 3*A*); while tree cover had a negative effect, total green space had a positive effect. Changes within the coniferous forest biome were driving this divergent response (Fig. 3*B*). In contrast, both total green space and tree cover in particular had negative effects on PM concentrations in the savanna/grassland biomes, albeit with greater variation in their effect as indicated by 95% CI.

**Fig. 4.**
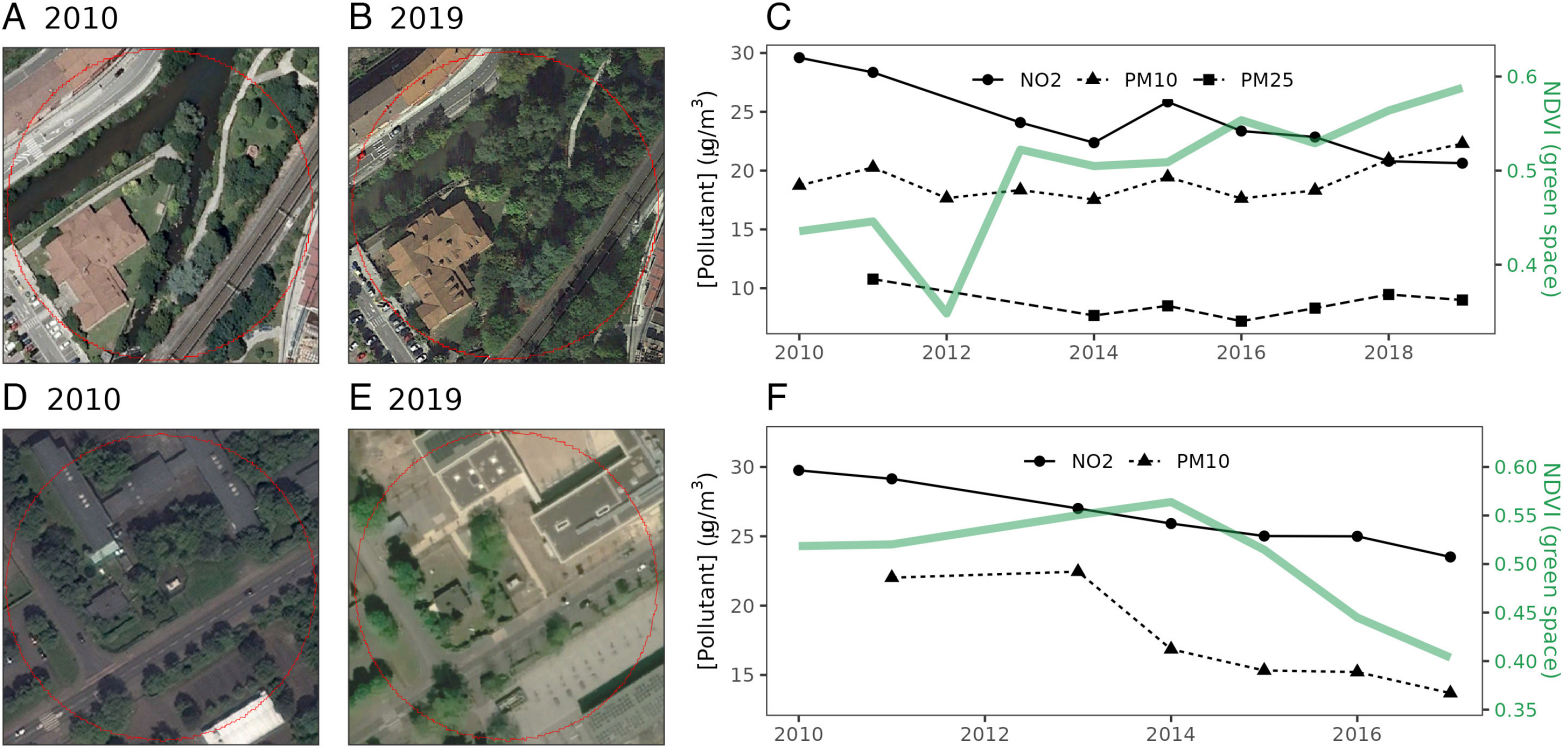
Example of an extreme increase (*A*−*C*) and decrease (*D*−*F*) in green space within a 60-m buffer (street-level) of two air quality–monitoring stations. Aerial photographs from Google Earth Pro shown for reference.

**Fig. 5.**
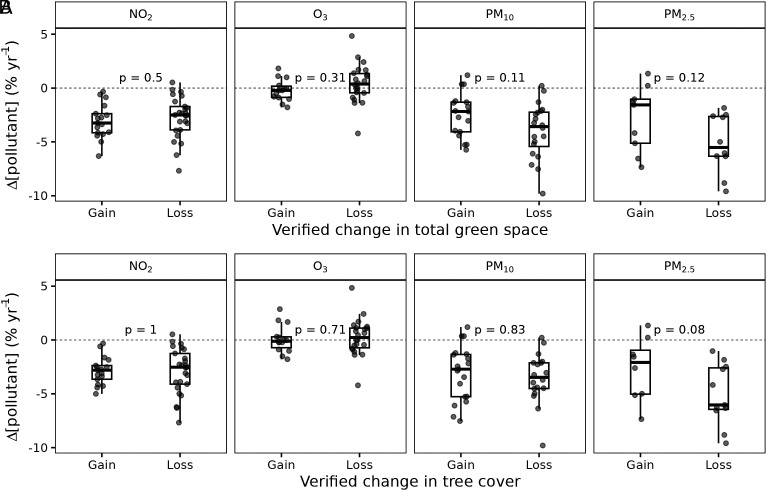
Changes in air pollutant concentrations at street-level (within 60 m) between 2010 and 2019 for a sub-set of air quality stations with the greatest gains (n = 37) and losses (n = 65) in green space (*A*) and tree cover (*B*) over the same period. *P*-values derived from linear mixed-effects models are shown to indicate no significant difference (*P* = 0.05) in air pollutant changes between stations with extreme gains and losses of green space.

To test the robustness of our finding that there is little effect of street-scale changes in green space on air pollutant changes, we performed a manual verification exercise of the satellite-derived NDVI time series. We screened the green space changes at a subset of stations within 60 m of air quality monitors. We first ranked stations by the magnitude of their trend in NDVI to identify candidate stations for manual screening. Then, we used visual interpretation of historical satellite imagery in Google Earth Pro (examples in Fig. 4) to produce a sample of 37 stations with verified gains in green space extent (mean gain of 13.7% total cover and 8.3% tree cover in particular) and 65 stations with verified green space losses (mean loss of 17.4% total cover and 6.7% tree cover). We found no significant difference in pollutant concentration changes between stations with gains and losses of total green space (Fig. 5*A*) or tree cover in particular (Fig. 5*B*). This lack of green space effect was consistent across all pollutants, thereby corroborating the results from our satellite-based statistical models. The result aligns with findings from local-scale experiments and modeling work demonstrating that planting vegetation, especially tall vegetation, close to emission sources such as along roadsides can reduce microscale ventilation and consequently exacerbate ambient pollution levels (9, 16, 32). Site conditions largely dictate the aerodynamic effect of green space on air quality at local scales. For example, aerodynamic effects can be beneficial in terms of channeling pollutants away from pedestrians (15). In cases where there are detrimental aerodynamic effects, however, the dispersion effect of urban green space can often be considerably stronger than the pollutant removal capacity of vegetation via deposition (16).

### Study Limitations.

We acknowledge that our analysis is purely correlative and that we cannot elucidate causal links between air quality and its drivers. Nevertheless, when placed in the context of experimental and modeling studies which do not find consistent effects of vegetation on air quality, our results suggest the causal links are scale- and context-specific and not widely generalizable. In addition, we attempted to control for potential confounders in our statistical models including anthropogenic emissions and climatic variations. With higher resolution emissions data such as road-specific traffic time series, we might expect to explain more of the temporal variation in air pollution concentrations. Similarly, the detail captured by the satellite data we used was limited, and thus we could not characterize spatio-temporal variation in vegetation characteristics such as shape, size, and porosity which are important determinants of air pollutant deposition and dispersion (16, 33). Further, topography and urban form play a key role in associations between wind speed and street canyon ventilation. In our study, we did not have the data to quantify changes in urban form surrounding air quality stations. Apart from microscale determinants of air quality, we could not incorporate macroscale dynamics that operate at regional scales such as long-range transport of aerosols, particularly for Southern Europe.

The satellite time series used to quantify green space change in our analysis may be subject to temporal biases caused by image artifacts like cloud contamination or atmospheric interference (34). To mitigate these artifacts, we performed rigorous satellite data pre-processing and manually verified green space changes at a subset of sites using visual interpretation of aerial imagery. The correspondence between samplers was 84% for changes in total green space and 85% for changes in tree cover (*SI Appendix*, Table S1). Finally, it is important to note that the absolute pollutant concentrations in our study are low compared to those found in India and China. Therefore, it is possible that a greater vegetation effect on air quality emerges at substantially higher pollutant concentrations than the range that was captured in our data.

### Implications and Conclusions.

Our findings add important nuance to the assumption generally held in the literature and public discourse that green space improves air quality. We found that the extent to which green space ameliorates air quality varies widely between green space type (tree cover versus total green space), spatial scale, and biome. Recent reviews of the literature have identified a significant knowledge gap regarding how green space impacts on air pollution vary across a range of spatial scales (10, 32). We found that total green space had no significant effect at street-level, yet at borough to city scales, tree cover in particular was associated with declines in O_3_, PM_10_, and PM_2.5_. This aligns with theory and modelling work which suggest that at local scales, dispersion and aerodynamic mechanisms are dominant and may act to exacerbate air pollution. However, at regional scales, deposition mechanisms are dominant and act to mitigate air pollution (10, 16). Indeed, in a review of experimental and modeling literature, the presence of vegetation in street canyons led to an average rise of 20 to 96% in pollutant concentrations when compared to street canyons lacking vegetation (9). At city and regional scales, modeling studies estimate that green space can ameliorate air quality via deposition, albeit at slow rates of between 1 and 2% over decadal time frames (10, 35–37). In our analysis spanning two continents, we found even smaller effects of green space—0.8% decrease in pollutant concentrations over 10 y—when averaging over spatial scales.

Authors routinely portray the role of urban vegetation in reducing air pollution as a central ecosystem service in regulating urban environments (38), and the most cited economic benefit of urban trees (39). These widely held assumptions have resulted in urban vegetation and air quality featuring prominently in proposals for generating environmental economic accounts, including the UN System for Environmental-Economic Accounting accounts for urban areas (14). Modeling tools which focus on deposition [e.g., i-Tree Eco (40)] and those that focus on dispersion (e.g., fluid dynamic models reviewed in ref. 41) are often used to account for the beneficial effects of urban vegetation on air quality. While models used in ecosystem accounting like i-Tree Eco estimate air quality improvement with a tree-centric deposition model, other models calculate pollutant capture based on estimated deposition velocities of broadly defined land cover categories (42). Nonetheless, proponents of deposition models contend that the absolute volumes of deposition on tree surfaces translate into “substantial health benefits” (37). Our results provide further evidence to a growing consensus that i-Tree Eco and similar models may substantially overestimate both the effect urban vegetation has in providing air quality improvements and corresponding human health values, particularly when interpreted at local scales (43).

Several issues raise questions about the accuracy and generalizability of deposition models which do not account for the dispersion dynamics introduced by vegetation at the street-level. First, these models do not account for the spatial context of vegetation in terms of its spatial configuration and proximity to other urban infrastructure which can act to reduce or increase ventilation. Such ventilation and dilution effects have demonstrably greater capacity to reduce pollution concentrations than deposition (16, 44). Second, deposition models do not account for any aspects of pollution dispersion or potential feedback between processes that respond to changes in atmospheric chemistry and wet deposition (35). Elevated levels of dry deposition on vegetation can reduce wet deposition enough to substantially reduce or nearly negate dry deposition effects, particularly for slowly depositing compounds like PM_2.5_. Third, the parameters for deposition velocity used in i-Tree Eco and similar models are generated from a small number of studies involving conditions that may not generalizable to other regions or tree species (15).

Given the variation in street-level vegetation effects on air quality, and the overall minor effect size (−0.8% over 10 y) observed in our study, there is reason for skepticism about the estimated health benefits that urban green cover supposedly generates via improving air quality. In their interdisciplinary review, Eisenman et al. (43) highlight a conspicuous lack of empirical support for the purported links between urban green cover and positive effects on human health—such as a lower prevalence of asthma. With only one exception (45), all population-based empirical human health studies covered in their review either contradict or fail to support the purported benefits of urban trees in reducing asthma. Many investigations either find no link between urban tree cover and childhood asthma (46, 47) or find that areas with increased tree pollen can lead to higher seasonal peaks in emergency department visits, hospitalizations and allergy medicine prescriptions among both children and adults (48). In contrast to using mechanistic models of the biophysical interactions of trees and air pollution levels, the epidemiological research linking trees to respiratory illness highlights negative effects of trees’ pollen production—not the benefits of pollution reduction (43).

While our results do not support the hypothesis that the vegetation effects on air pollution would lead to substantial public health benefits, there are still many important reasons for including trees in urban environments. Trees and other urban greenery are vital components of urban ecosystems and biodiversity. They ameliorate extremes in urban area hydrology (both flooding and drought) and contribute to microclimate regulation. Through reducing the heat-island effect, trees can reduce urban residents’ mortality from extreme temperatures (49). Urban green spaces can also promote physical activity, and improve mental health outcomes such as mood and mental attention, which ultimately improve community wellbeing (50, 51).

In light of the evidence that urban trees may, in certain configurations, actually worsen air quality, Vos et al. (16) propose a paradigm shift, moving from asking “*How to use urban vegetation to improve local air quality?*” to “*How can urban vegetation be used without significantly deteriorating the local air quality?*”. Recent reviews of the literature (9–12, 15, 17, 32, 52) show that vegetation performs best when it has a limited impact on decreasing airflow. For example, dense rows of trees without gaps and with closed canopies should be avoided in street canyons with high traffic. In more open areas with greater airflow, vegetation can serve as a barrier between an emission source and pedestrians but not as an air filtration device. Other guiding principles for road-side vegetation include: i) low-level vegetation like hedges are best for street canyons so long as they are continuous, dense (low-porosity), and preferably centrally located as opposed to alongside the street; ii) mid- to high-level vegetation like trees are appropriate for open road environments (i.e., outside street canyons) so long as they are dense and continuous acting as barriers between the road and surrounds. Urban planners should also consider whether the tree species may be capable of producing large amounts of either pollen or biogenic VOC that can decrease air quality. Physical barriers constructed from non-vegetative material may also be effective at mitigating air pollution if the above guidelines are followed. However, if the focus is on large-scale air pollution abatement with significance for public health, our results suggest that post-emission strategies such as urban greening are inadequate solutions. By continuing to promote vegetation’s purported capacity to mitigate pollution, we risk diverting resources from the most important strategy for improving air quality: reducing anthropogenic emissions.

## Materials and Methods

### Air Pollution Data.

We collected historical NO_2_, PM_10_, PM_2.5_, and O_3_ daily time series from air quality stations managed by the United States EPA (53) and the EEA (54) between 2010 and 2019. We chose air pollutants that are most often cited as significant risk factors for negative public health outcomes (1, 55) and that are most commonly used in studies exploring the effects of vegetation on air quality (10, 56). The EEA dataset contained 6,569 stations and the EPA dataset 2,871 stations that recorded concentrations for our selected pollutants during the study period. We used the Global Human Settlement Layers published by the European Commission (57) to identify stations located within urban and suburban areas. Here, urban was defined as 1 × 1 km grid cells with a density of built-up surface greater than 50%, and a density of at least 300 inhabitants and a minimum total population of 5,000. The sample sizes after filtering for urban stations were 4,791 for EEA and 1,421 for EPA. To better characterize the variation in urban context covered by the stations, we used OpenStreetMap data to calculate the distance to the closest road and the building footprint within a 30 m radius for each station.

Station time series were first aggregated to monthly averages. From the monthly time series, we derived annual averages and maximums, given that we were interested in how green space is associated with inter-annual changes in air pollution. Although annual averages allow us to explore the cumulative effect of green space throughout the year, the annual maximum allows us to test whether green space might have a larger effect size on months with peak pollutant concentrations. Raw pollutant time series were iteratively aggregated from hourly to daily to monthly and finally annual increments using a data exclusion criterion of 25% for each temporal increment as applied by the EEA and Solberg et al. (58). The data exclusion criterion meant that a time period was excluded if there were more than 25% missing readings during that period. Similarly, we excluded station time series with more than 3 y of missing data between 2010 and 2019 (~25% of the 10-y time series). Following the time series filtering, our dataset included 2,127 EEA stations and 488 EPA stations, resulting in 5,558 unique pollutant time series from 2,615 stations.

### Urban Green Space Data.

We derived two satellite-based measures of green space including the NDVI (59), and fractional tree cover. The former allows us to explore changes in green space in general, while the latter focusses on tree cover in particular. NDVI is a good proxy for fractional vegetation cover, and its temporal dynamics correspond to changes in vegetation structure and productivity over time (27). Trees are the object of most air quality mitigation studies and therefore changes in tree cover are expected to have the largest effects on pollutant concentrations relative to grass or shrubs (16, 37). We calculated NDVI from the Landsat satellite archive at 30 m resolution and fractional tree cover from the Terra MODIS Vegetation Continuous Fields dataset produced by the United States Geological Survey at 250 m resolution (60). Satellite data were extracted for a range of buffer zones (between 15 m and 16,000 m radii) around each air quality station between 2010 and 2019. Due to the limited spatial resolution of the MODIS tree cover data, and given no other high-resolution tree cover time series data are currently available, we were only able to extract tree cover changes for buffer sizes greater than 120 m. The range of buffer zones was chosen based on the most common spatial scales employed in air pollution dispersion models in the literature (52). We further categorized the zones into spatial scales of street-level (15 to 60 m), borough-level (120 to 1,000 m), and city-level (2,000 to 16,000 m).

While the MODIS tree cover data were pre-computed, we generated NDVI from Landsat 7 and 8 surface reflectance images which were processed in the Google Earth Engine cloud-computing environment (61) to exclude cloud- and snow-covered pixels using the “pixel_qa” band. Given we were working across two Landsat sensors, we applied published cross-calibration coefficients to harmonize Landsat 7 and 8 reflectance values to ensure consistency in spectral responses over time (62). We calculated annual median NDVI values for each Landsat pixel within the station buffer zones around each station. The annual median was used to avoid undue influence from NDVI outlier values. The pixel values were averaged across each buffer zone for each year in the time series. The MODIS tree cover product is delivered as annual averages; therefore, no temporal aggregation was necessary.

To supplement the large-scale NDVI and tree cover analysis, we conducted a manual screening for extreme changes in green space where one would expect to find the largest effects on air quality. To do, this we calculated the linear trend in NDVI for each air quality station between 2010 and 2019. Candidate stations were ranked according to the magnitude of change in NDVI, and 125 stations with the greatest increases and decreases in NDVI were chosen for manual screening. Candidate stations were randomly split between five samplers (the authors of the paper) who used visual interpretation of Google Earth historical aerial imagery to estimate percentage changes in green space within a 60-m buffer of each station (see examples in Fig. 3). Green space was defined as any vegetated surface that was not cultivated including parks, road verges, green belts, trees, and green roofs. In addition to changes in total green space, the data collection protocol differentiated changes in trees versus grass/shrubs. We chose 60 m because it is an intermediate distance reported in the literature on microscale air pollution dispersion representing street-scale effects (52), and it was practical to estimate changes in green space through visual interpretation of aerial imagery. After filtering out stations where aerial imagery was missing in 2010 or 2019 or was too blurry to make an informed decision about green space change, we were left with a final sample of 37 stations with confirmed increases in green space and 65 with confirmed declines in green space.

To cross-validate the manual screening, green space changes at a random subset of 15 stations were interpreted by four of the same samplers. We quantified the correspondence between interpretations of changes in green space by grouping into three categories of stable (change < 10%), gain and loss (change >= 10%). We then calculated the balanced accuracy from a confusion matrix built from sampler-by-sampler interpretations (*SI Appendix*, Table S1). Balanced accuracy was defined as the average of the sensitivity (True Positive Rate) and specificity (True Negative Rate) and is expressed as a percentage.

### Emissions Data.

We used EDGARv6 global GHG (greenhouse gas) gridded anthropogenic emissions version 6 (63) as our source of data on anthropogenic GHG emissions worldwide—distributed by ECCAD-AERIS; Emissions of atmospheric Compounds and Compilation of Ancillary Data - An atmosphere Data and Service Centre (https://eccad3.sedoo.fr/metadata/601, accessed April 2023). Specifically, we collected annual emission data on NO_x_, PM_10_, and PM_2.5_ at a gridded spatial resolution of 0.1 × 0.1° from 2010 until 2018. NO_x_ emissions were used in NO_2_ and O_3_ models. We processed the data to aggregate emissions from all sectors. Time series were extracted for grid cells intersecting air pollution–monitoring stations. Unfortunately, 2019 data were not available from the original EDGARv6 dataset. To estimate emissions for 2019, we used a linear extrapolation method to estimate individual pollutants’ emission rates from 2013 until 2018. This was done at each air pollution–monitoring station. This allowed us to estimate emissions for 2019 based on the trend observed in previous years.

### Climate Data.

To control for and explore the effects of meteorological changes on air pollutant concentrations, we used the ERA5-Land climate reanalysis data from the Copernicus Climate Change services (64). ERA5-Land is a gridded dataset with a 10-km resolution. We extracted monthly means of air temperature 2 m above the ground, total precipitation, wind speed, and relative humidity for the grid cells intersecting the location of air pollution stations. Monthly time series were then aggregated up to annual averages for each station.

### Statistical Analysis.

The statistical analysis took place in three parts: i) We used linear mixed-effects models (65) to estimate the direction and magnitude of the changes in air pollutant concentrations over the entire set of air quality stations between 2010 and 2019. Year was included as a fixed-effect, while air quality station was assigned as a random effect in the model to control for any model-specific effects. Air pollutant concentrations were log-transformed to ensure residuals were normally distributed. ii) To quantify the association between green space and air pollution concentration over time while controlling for the effect of emissions and climate variables, we fitted the same model structure described above, except adding green space (NDVI and tree cover), emissions, and climate covariates as fixed-effects. Separate models were fit for each biome and each buffer zone used to aggregate green space around the stations. iii) The manually screened stations with extreme NDVI changes were used to fit mixed-effects models to test whether air pollution declines were greater at stations with gains in green space than at stations with losses in green space. Linear trends in climate variables and a categorical gain/loss in green space were included as fixed effects, while sampler was included as a random effect to control for any sampler-specific biases in interpretation of aerial imagery.

## Supplementary Material

Appendix 01 (PDF)

## Data Availability

Code supporting the analysis: https://github.com/zanderVenter/greenspace-air-pollution. Previously published data were used for this work and are cited in the methods section.
