# Supplementary material for: Reassessing the role of urban green space in air pollution control
Source: Proc Natl Acad Sci U S A. 2024 Jan 29;121(6):e2306200121. doi: 10.1073/pnas.2306200121 (PMC10861851; doi:10.1073/pnas.2306200121)
Supplement: Supplementary file 1 — Appendix 01 (PDF) [file pnas.2306200121.sapp.pdf]

Supplementary information

Table S1. Cross-validation between four samplers (S) interpreting very high resolution aerial imagery in 2010 and 2019 to estimate changes in green space within 60m of air pollution monitoring stations (n = 15). Correspondence between change estimates for each unique combination of samplers are shown as class-wise balanced accuracies from a confusion matrix.

Variable measured	Change category	S1 ~ S2	S1 ~ S3	S1 ~ S4	S2 ~ S3	S2 ~ S4	S3 ~ S4	Average
Green space cover	Gain	100	75	100	75	100	100	92
	Loss	89	85	70	80	65	70	76
	Stable	88	88	65	87	60	75	77
Tree cover	Gain	100	50	100	50	100	100	83
	Loss	96	88	93	75	96	89	90
	Stable	96	80	93	70	96	86	87

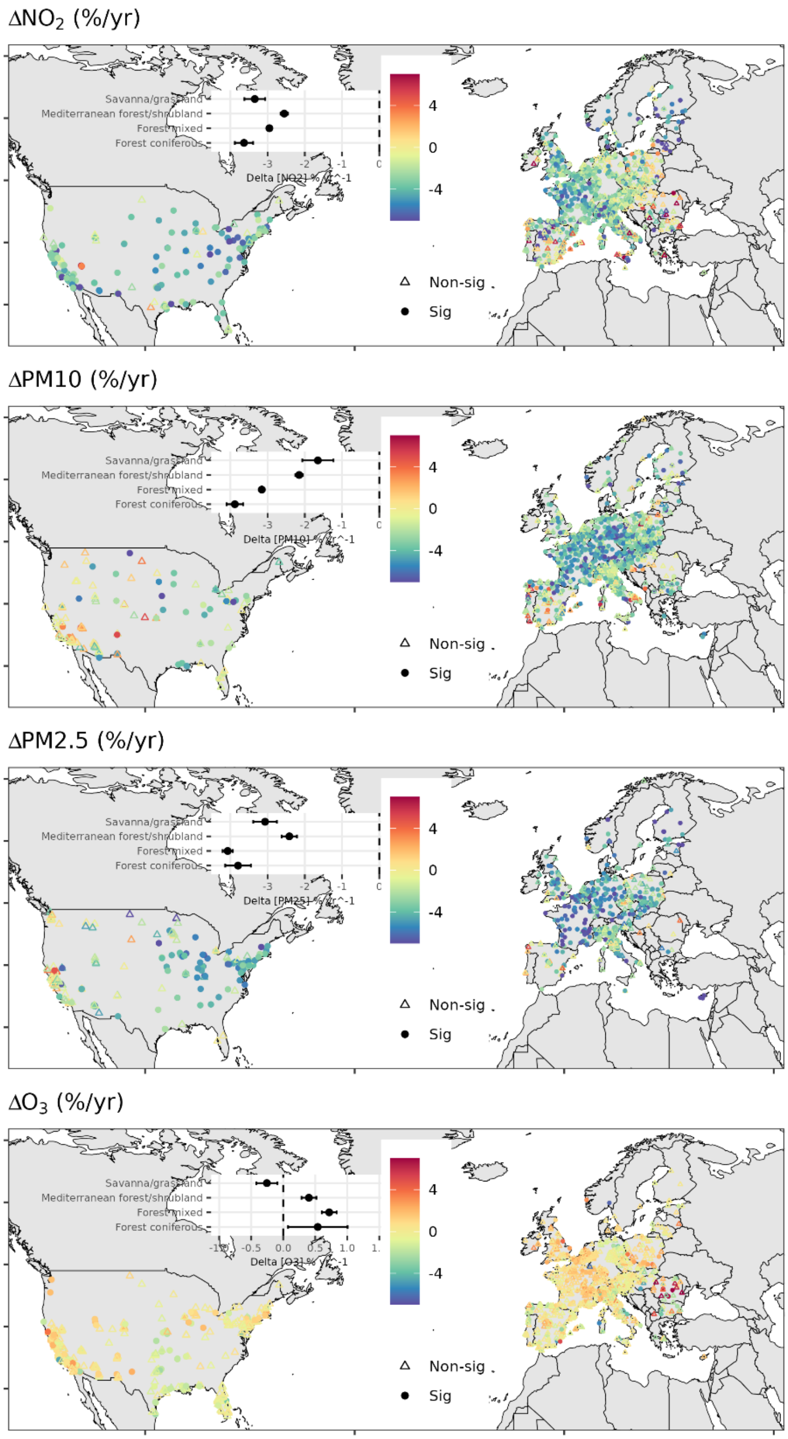

Figure S1. Spatial distribution of the linear trends in air pollutant concentrations. Points are colored by the magnitude of the coefficient from linear regressions of annual median air pollutant concentrations on year. Significant trends ($p < 0.05$) are indicated with solid points, while non-significant trends are hollow triangles. Inset plots show mean trends (points) and standard errors (error bars) for stations within each biome.

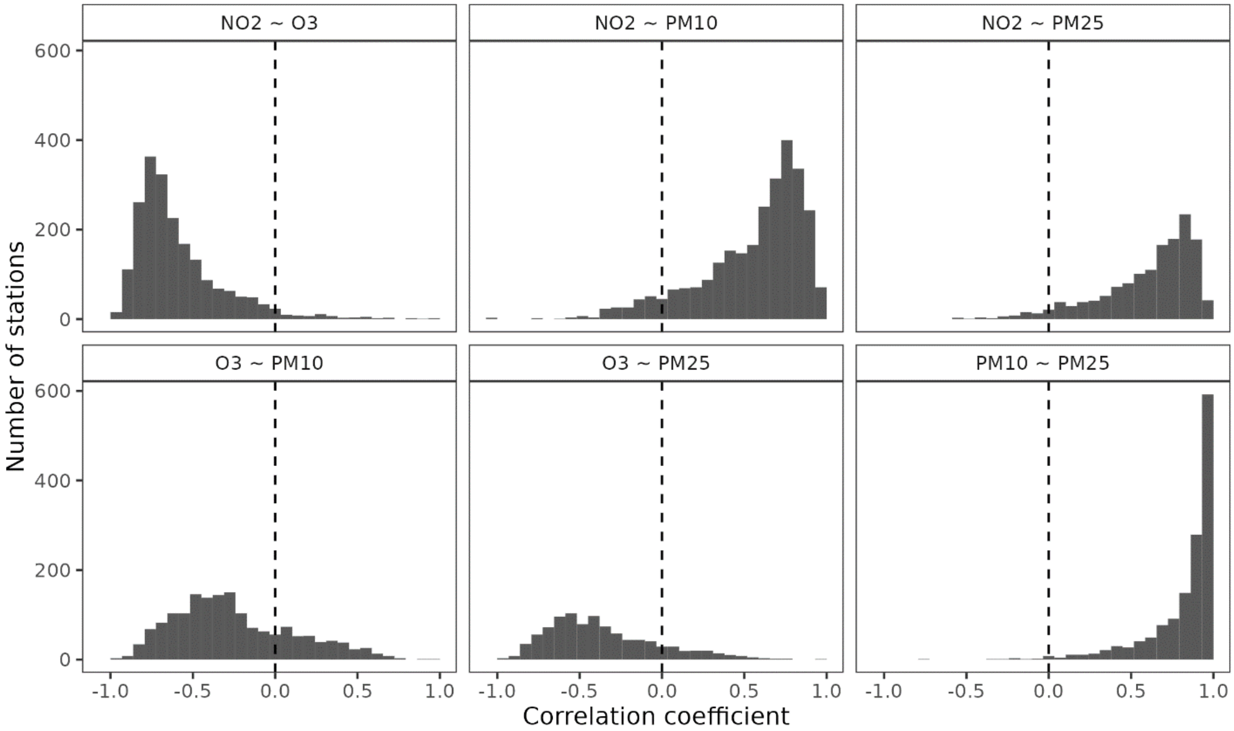

Figure S2. Distribution of correlation coefficients between air pollutants at the station level. We calculated Pearson's correlation coefficients between each combination of air pollutants for each monitoring station. Stations may only contain a subset of all four air pollutants measured and therefore some histograms have a smaller sample size.

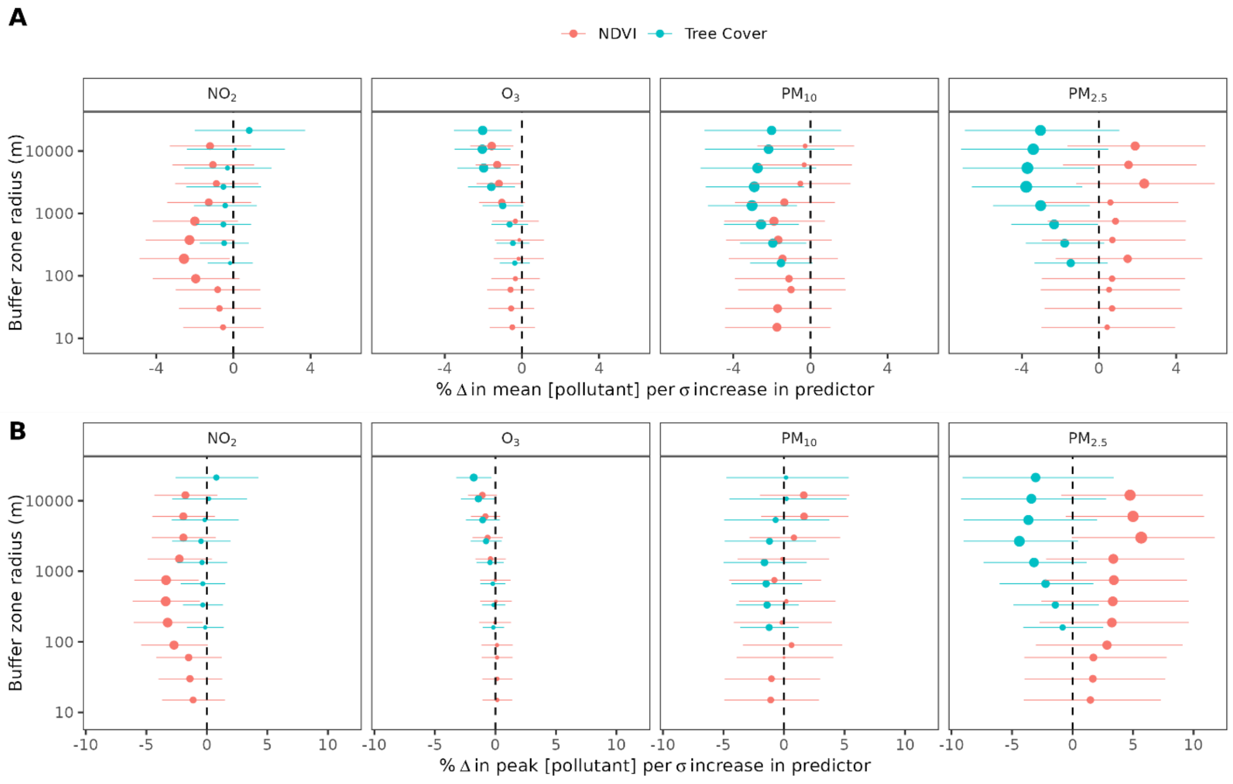

Figure S3. Estimates of the association between green space and air pollutant concentration changes for different buffer zone sizes. Associations with mean annual (A) and peak annual (B) air pollutions are shown. The buffer zone defined the circular area around the air quality station within which green space was averaged per year. Estimates (points) and 95% confidence intervals (error bars) are from linear mixed-effects models and are expressed as percentage changes in air pollutant concentrations per standard deviation (δ) increase in NDVI or tree cover.
